# Practical Application of Deep Learning in Diagnostic Neuropathology—Reimagining a Histological Asset in the Era of Precision Medicine

**DOI:** 10.3390/cancers16111976

**Published:** 2024-05-23

**Authors:** Katherine Rich, Kira Tosefsky, Karina C. Martin, Ali Bashashati, Stephen Yip

**Affiliations:** 1 Bioinformatics Graduate Program, University of British Columbia, Vancouver, BC V6T 1Z4, Canada; richkath@student.ubc.ca; 2 Faculty of Medicine, University of British Columbia, Vancouver, BC V6T 1Z4, Canada; kiratos@student.ubc.ca (K.T.); karina.chornenka@vch.ca (K.C.M.); 3 Department of Pathology & Laboratory Medicine, University of British Columbia, Vancouver, BC V6T 1Z4, Canada; 4 School of Biomedical Engineering, University of British Columbia, Vancouver, BC V6T 1Z4, Canada

**Keywords:** neuropathology, deep learning, artificial intelligence

## Abstract

**Simple Summary:**

Technological and scientific innovations, from genetic sequencing to digital pathology slide scanners, have drastically altered the field of neuropathology. The rapid development of deep learning, and its widespread adoption in multiple industries suggest it may be the cause of the next big shift. In this review, we investigate how deep learning has been utilized in neuropathology thus far. We explore how common machine learning frameworks have been applied in the past and how they may be used in the future. Finally, we look at the challenges and risks of the widespread adoption of deep learning models in clinical practice.

**Abstract:**

In the past few decades, neuropathology has experienced several paradigm shifts with the introduction of new technologies. Deep learning, a rapidly progressing subfield of machine learning, seems to be the next innovation to alter the diagnostic workflow. In this review, we will explore the recent changes in the field of neuropathology and how this has led to an increased focus on molecular features in diagnosis and prognosis. Then, we will examine the work carried out to train deep learning models for various diagnostic tasks in neuropathology, as well as the machine learning frameworks they used. Focus will be given to both the challenges and successes highlighted therein, as well as what these trends may tell us about future roadblocks in the widespread adoption of this new technology. Finally, we will touch on recent trends in deep learning, as applied to digital pathology more generally, and what this may tell us about the future of deep learning applications in neuropathology.

## 1. Introduction

Recent years in the field of diagnostic neuropathology have shown an increasing reliance on molecular testing to yield accurate diagnosis and classification, particularly in the context of brain tumor diagnoses since the release of the 2021 WHO Classification of Central Nervous System (CNS) Neoplasms [1]. Nonetheless, the morphological assessment of histopathologic slides remains the cornerstone of tissue diagnosis, as it serves as the foundation upon which all further ancillary testing is based. Histopathologic assessment is typically accomplished by pathologists with subspecialty training in neuropathology. However, with the rapid advancement of the field, there has been an increasing interest in utilizing artificial intelligence (AI) methods to aid neuropathologists in providing accurate assessments of biopsied tissue in a maximally cost-effective and efficient manner. The application of these methods may limit the use of unnecessary resources such as additional immunohistochemical stains to narrow differential diagnoses, thereby decreasing turnaround times to optimize patient care. The aim of this review is to summarize the recent advancements in diagnostic neuropathology and the application of AI methods to tissue diagnosis, as well as to discuss some of the benefits and challenges of applying AI models to diagnostic neuropathology.

## 2. History of Glass-Based and Molecular Features in Brain Cancer

Brain cancer diagnosis and grading have historically relied on the visual inspection of hematoxylin and eosin (H&E)-stained glass slides [2]. This assessment typically establishes a morphological differential diagnosis that determines the diagnostic workup to follow, such as exposing the tissue to special stains and immunohistochemistry (IHC). The results of these ancillary tests help the neuropathologist narrow the differential and arrive at an accurate diagnosis. However, interobserver variability and inconsistent relationships between histopathologic features and patient outcomes have been increasingly recognized as notable limitations to the exclusive reliance on this approach [3,4]. Advances in the understanding of the molecular underpinnings that drive various brain neoplasms led to the introduction of molecular diagnostic criteria in the 2016 WHO Classification of Central Nervous System (CNS) Tumors [1,5]. One such example is showcased in the seminal works by Parsons et al. [6] and Yan et al. [7], which demonstrated that diffuse gliomas harboring mutations in *IDH1* and/or *IDH2 (IDH1/2)* were predictive of a less aggressive clinical course than *IDH*-wildtype tumors. As such, tumors with *IDH1/2* mutations designated a diagnosis of either *IDH*-mutant astrocytoma or *IDH*-mutant secondary glioblastoma multiforme, depending on the morphologic characteristics of the tumor [5]. Additionally, the presence of an *IDH1/2* mutation in combination with 1p19q co-deletion became diagnostic of oligodendroglioma, irrespective of whether the neoplasm demonstrated astrocytic or oligodendroglial morphologic features [5,8]. The WHO 2016 classification scheme also introduced major changes in the classification of embryonal tumors—medulloblastomas were separated into four molecular subtypes based on *WNT*, *SHH*, and *TP53* statuses; *SMARCB1/SMARCA4* loss was introduced as a necessary criterion for the diagnosis of atypical teratoid/rhabdoid tumor; and embryonal tumors with multilayered rosettes with C19MC amplifications was introduced as its own diagnostic category [5,8].

The concept of the integrated “histomolecular” diagnosis was expanded to apply to several other tumor entities in the WHO 2021 update. In this edition, a major revision was the distinction of pediatric-type diffuse gliomas from adult-type diffuse gliomas, due to their unique molecular signatures [1]. Within the adult-type diffuse glioma classification, the significance of a neoplasm harboring an *IDH* mutation evolved from the WHO 2016 classification, now denoting either an *IDH*-mutant astrocytoma or *IDH*-mutant oligodendroglioma, dependent on the 1p19q status. As such, the concept of an *IDH*-mutant glioblastoma became obsolete, as the prognostic implications and underlying molecular alterations of *IDH*-mutant and *IDH*-wildtype gliomas were shown to be distinct [9,10,11]. Amongst *IDH*-mutant gliomas, the homozygous deletion of *CDKN2A/B* has been shown to be an important marker for adverse clinical outcomes and this has been reflected in the updated grading scheme of the IDH-mutant astrocytoma [1]. A summary of the morphologic and molecular diagnostic and grading criteria of adult-type diffuse gliomas is outlined in Table 1.

Many molecular alterations including and beyond those used in classification criteria have demonstrated prognostic relevance (e.g., *IDH*-mutation status or *WNT* molecular subgroup of medulloblastoma), while others such as *MGMT* promoter hypermethylation have been shown to predict response to therapy [12]. More recently, characterizing the epigenomic signature of brain tumors through comprehensive DNA methylation profiling has emerged as an additional tool for diagnosis and subclassification beyond an entity’s histopathologic and genomic characteristics [13]. In the workup of some CNS tumors, this serves as an additional tool to arrive at an accurate diagnosis, while in other tumor entities, such as high-grade astrocytoma with piloid features (HGAP), DNA methylation profiling serves as the key method in confirming its diagnosis [1].

## 3. Current Diagnostic Challenges and Shortcomings

Given the significance of molecular pathology in the classification of CNS tumors as outlined above, clinical neuropathologic diagnosis has come to heavily rely on advanced molecular techniques such as chromosomal microarrays, next-generation sequencing, and methylation profiling, with single-gene sequencing, fluorescence in situ hybridization, targeted methylation assays and immunohistochemical analyses serving as imperfect substitutes for more advanced methods [14]. Integrating molecular features with morphologic characteristics represents an ongoing challenge, given the current reliance on neuropathologists, for the evaluation of individual H&E slides for morphologic evaluation. Deep learning (DL) analysis of digitized whole-slide images (WSIs) provides an opportunity to efficiently integrate molecular pathology with unbiased assessments of histopathologic features [15].

Beyond efficient morphologic assessment, DL algorithms have been shown to identify patterns in WSIs, which may, themselves, be used to predict molecular alterations both within neuropathology [16,17] and other pathology disciplines [18,19]. This capability may be harnessed to assist with subtype classification and prognosis in resource-limited settings, as the availability and accessibility of costly advanced molecular diagnostic technologies varies greatly within and between countries [20].

## 4. Supervised Machine Learning

Over the past decade, machine learning has undergone rapid development and evolved into several distinct frameworks of deep learning models. Briefly, deep learning is a subfield of machine learning, which involves the use of multiple artificial neural layers designed to mimic the structure and interaction of human neurons. It is characterized by its ability to automatically learn features from input data and has a much larger model size. However, the terms ‘deep learning’, ‘machine learning’, and ‘artificial intelligence’ tend to be used interchangeably outside of technical discussion.

The widely agreed-upon start-point for this progress is the introduction of the ImageNet dataset in 2009 [21], which led to the breakthrough in image classification by Krizhevsky et al. [22]. This achievement also made it clear that, for supervised AI models, performance is highly dependent on the dataset it is trained upon and any limitations of the training data will be reflected during model inference. This makes the development of models for use in diagnostic neuropathology challenging. As neuropathology covers an expansive group of conditions with highly heterogeneous morphologies, any model aiming to be used in standard clinical practice would need to be able to, *at a minimum*, identify outliers and thus be exposed to them in some form during training. Even when models are specifically tuned to specific neoplasms, such as gliomas, they still need to be able to account for the inherent morphological heterogeneity, as well as variance from different staining protocols and scanners. This is part of the reason why developing models for neuropathology is so challenging. The creation of large, high-quality datasets that can meet these requirements is limited by privacy and storage considerations, the need for image labeling by expert neuropathologists, and the rarity of certain CNS tumor types [23].

Given this, it is unsurprising that the focus of most studies applying supervised AI to neuropathology focus on diffuse gliomas, as there is a large publicly available dataset of hundreds of cases provided by The Cancer Genome Atlas of low-grade glioma and glioblastoma (LGG and GBM) [23]. Over the past decade, several studies have focused on predicting tumor grade or histologic subtype from the TCGA datasets [24,25,26]. A notable drawback to this approach is the dependence on the diagnostic framework provided by the WHO classification for the labels of the training dataset. Other than Jose et al. [27], all the aforementioned studies were published prior to 2021 and, thus, preceded the updates introduced by the CNS WHO 2021 classification, in which molecular testing is the cornerstone of many tumor diagnoses. While the previous diagnostic framework is still similar enough to be of clinical use today, the evolution of the field of neuropathology seems to be continuing on a trajectory of a seemingly ever-increasing reliance on molecular diagnostics. Future CNS WHO updates could make the models developed in these earlier studies lose relevance.

Taking an alternative approach, several studies applied deep convolutional neural networks (most commonly the ResNet architecture [28]) to the same TCGA datasets to predict clinical outcomes directly from H&E-stained WSIs [25,29,30]. They were thus able to avoid dependence on the WHO diagnostic framework. Other research approaches integrated additional datatypes to improve the performance of their model. Rathore et al. [31] combined features from pre-operative MRI scans and H&E-stained WSIs of 107 glioblastoma cases (orig. TCGA-GBM) to classify long-term and short-term survivors (AUC 0.80). Other studies, such as the one by Chen et al. [32], utilized non-imaging data by combining the genomic data in the TCGA datasets to augment their multimodal network. They achieved a c-index of 0.808 on the TCGA-LGG cohort.

Due to the diagnostic relevance of *IDH* mutations in adult-type diffuse gliomas, Liechty et al. [33] trained a DenseNet-121 convolutional neural network (CNN) model to predict *IDH* status using the TCGA datasets. While there was a significant drop in accuracy from the TCGA datasets to a separate internal dataset (AUC 0.988 vs. 0.829), their model achieved similar diagnostic accuracy to expert pathologists; however, the mistakes the pathologists and the model made were distinct. Hewitt et al. designed a weakly supervised DL pipeline to predict glioma subtype by utilizing both 2016 and 2021 WHO classification schemes and predicting the core molecular alterations—*IDH* mutation, 1p19q codeletion, and *ATRX* mutation (AUC 0.90, 0.79, & 0.87) [16]. Notably, this work used a large dataset (n = 1882) from BRAIN UK [34]. The recent introduction of this resource, as well as that of the Digital Brain Tumour Atlas [35], which both contain a much wider variety of both neoplasms and other CNS diseases in comparison to TCGA, will likely lead to a greater prevalence and diversity of research in the field of digital neuropathology.

Research in neuropathology using supervised AI outside of low-grade gliomas and glioblastomas is limited, with meningiomas coming in a distant third place. Gennatas et al. [36] used demographic, clinical, pathologic, and radiographic data to predict the clinical outcomes of meningioma cases using a dataset of 257 neoplasms from 235 patients. Taking a different approach, Sehring et al. instead focused on classifying clinically relevant methylation subtypes from histologic slides [37]. Perhaps the most impressive utilization of supervised AI for classifying CNS neoplasms in general was the work carried out by Capper et al. [38], who presented a DNA-methylation based classification model that was trained and tested on a dataset spanning the approximately 100 tumor types of the CNS across various age groups.

Another limitation to the above methods is that the models are designed to take in post-surgical images of fixed samples. This makes them unable to assist in clinical decision-making during tumor resection. To address this, several studies have investigated the application of machine-learning to intraoperative samples. Abas et al. [39] presented a method of classifying neoplastic vs. non-neoplastic astrocytes. Following this, Orringer et al. [40] introduced an image acquisition and processing method referred to as stimulated Raman histology that can generate virtual H&E-stained slides and used it to train a brain-subtype classifier. This technology was later used by Hollon et al. [41,42] and Reinecke et al. [43] to predict diagnosis at the bedside in near-real time in an automated fashion.

As we have highlighted in this section, the main challenge of applying supervised AI models in neuropathology is the need for large and labeled datasets. While the most straightforward solution may be to simply obtain more data. The cost per pathology image is exponentially more than images in other machine learning domains; this is due not only to the requirement of specialized scanners to capture WSIs, but also to the expert knowledge needed to generate labels for these images. Moreover, as neuropathology encompasses many rare neoplasms, acquiring an adequate number of cases for each entity to build a sufficient dataset seems like an inconceivable task. Therefore, while data collection may not be a feasible solution, several innovations in the field of machine learning could provide tools to augment the data already available at hand or algorithms that can learn on unlabeled data.

## 5. Generative Machine Learning

Generative AI is a comparatively recent development in machine learning, where the model is designed to produce, or ‘generate’, data. This data can be in the same format as the original data domain or an alternative, related domain (e.g., text-to-image generators). The two most popular models for synthetic image generation are Generative Adversarial Networks (GANs) and the more recently introduced Diffusion probabilistic models, each with their own unique benefits and drawbacks. GANs are rather notorious for their training difficulty, often suffering from a phenomenon known as ‘mode collapse’, where the image generator will learn to only produce a limited number of synthetic images that meet the training requirements [44]. On the other hand, diffusion models are a newer development and are, thus, relatively untested in the field of digital pathology. Additionally, they are much more computationally expensive than GANs during both training and inference [45].

In the field of digital pathology, the most common application of this technology is to generate synthetic images of pathology slides. These can then be used in downstream tasks, including, but not limited to, augmenting training data for AI models, producing de-identified data to alleviate patient privacy concerns, or education. However, with the recent rise of large language models (LLMs), such as ChatGPT, applications for text generating models have also begun to be considered in a clinical setting.

The use of generative AI in neuropathology is in its infancy, but studies to date demonstrate several potential applications. Levine et al. used a type of GAN model (ProGAN) to generate synthetic images from the TCGA-LGG dataset (among other cancer types) and had the images assessed by experienced pathologists [46]. Not only were the pathologists able to identify the histotype of the images from a single patch, but the synthetic patches were also shown to improve the performance of a machine learning classifier, when used to augment the training set. Conversely, Moghadam et al. [47] trained a diffusion model on a subset of the TCGA-LGG dataset and compared their work directly with the results of Levine et al. [46]. It was found that the diffusion model outperformed the ProGAN model, both in their selected evaluation metrics (Inception Score, Fréchet inception distance, etc.) and on visual inspection.

As for text-based generative models, Truhn et al. used LLMs to extract structured information from the pathology reports of glioblastoma patients and demonstrated their ability to accurately extract relevant information [48]. However, it also highlighted some of the drawbacks of LLMs, most notably the limited input length and the inaccuracies related to handwritten annotations.

These studies demonstrate the potential of generative models in the field of neuropathology—both for image and text generation. With the rapid pace of development in this area of machine learning, we can expect even more applications of it being put forward in the coming years. As an example, we can expect models that are able to automatically generate a pathology report from histology slides.

## 6. Semi-Supervised and Self-Supervised Machine Learning

Recent years in machine learning research have demonstrated a push away from supervised methods and advances towards semi-supervised and self-supervised machine learning models. Briefly, semi-supervised models are those that use only some labeled data in conjunction with unlabeled data. This framework allows researchers to make use of archived data for which annotations or reports may be missing or otherwise unavailable. It can also address the need for labeled data, as discussed earlier. ‘Weakly supervised’ models are similar to semi-supervised models, where only slide-level labels are used. This is in contrast to supervised models, that have exhaustive annotations for each patch within the WSI. The majority of the studies previously discussed in Section 4 would technically qualify as ‘weakly supervised’ models. With the emerging capability of LLMs to analyze and extract information from pathology reports, this could enable a more automated data generation framework. Such tools could make weakly supervised models an invaluable tool for future for image analysis efforts.

**Figure 1:**
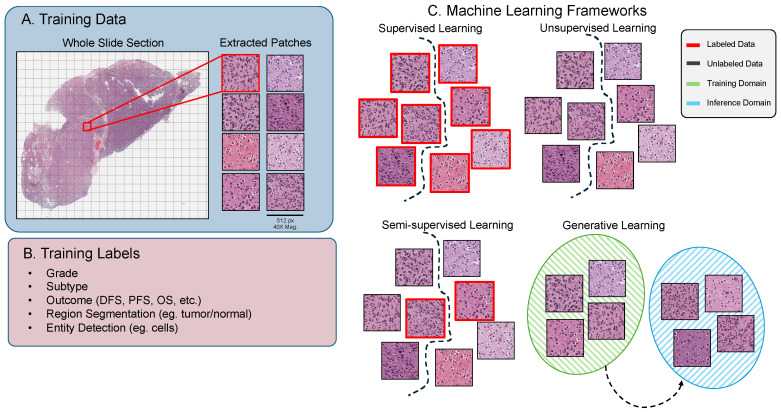
Overview of machine learning framework in digital pathology. (**A**) The commonly used method for gathering training data from H&E-stained histopathology images, where smaller patches are extracted from the gigapixel WSIs. (**B**) Examples of labels paired with histopathology image data, note the expertise required to generate this information. (**C**) Training frameworks used in machine learning that are frequently used in digital pathology contexts for a variety of different tasks.

Self-supervised models, on the other hand, are a subset of unsupervised models that generate implicit labels from the training data. This alleviates the need for labels, which, in the context of neuropathology, can be incredibly expensive both in direct cost (e.g., subjecting a sample to molecular testing such as genomic sequencing) or time (expert pathologic examination to determine the tumor subtype and grade), as they require specialized domain knowledge to produce. Foundational models are usually self-supervised models that are pretrained on vast quantities of data, with the end goal of producing weights that can be finetuned to achieve a state-of-the-art performance on a variety of tasks. In Figure 1, you can see a breakdown of the four types of machine learning models discussed thus far.

Though no foundational model has been trained that focuses solely on neuropathology, several general-purpose digital pathology models have been introduced. Huang et al. developed a visual–language model based on the OpenPath dataset (208,414 image pairs), which originated from medical twitter [49]. The model was able to retrieve relevant images from text input. CTransPath [50], Phikon [51], and Virchow [52] are all examples of recent foundation models trained on huge WSI datasets (>30,000 WSIs, >100,000 WSIs, and >1.5 million WSIs) that achieve great performance on downstream tumor-normal classification tasks (AUC: 0.954, 0.898, and 0.795, respectively). Chen et al. went a step further and demonstrated the capabilities of their model, UNI, in more domain-relevant tasks for neuropathology [53]. They reported a balanced accuracy of 85.6% for 2-class glioma *IDH1* mutation prediction, 56.2% at 5-class glioma subtyping, 88.3% at 12-class brain tumor cancer classification, and 67.5% at a 30-class brain tumor subtyping task.

## 7. Challenges and Risks

While DL poses many opportunities for streamlining and refining brain tumor diagnosis and prognosis, several challenges remain to be surmounted before widespread clinical use can be recommended. Digital pathology workflows involve sample delivery from hospital to pathology laboratory for sectioning, staining, and additional tests; scanning of slides; and storage in a server, at which point image analysis requests can be sent to pathologists [54]. Pathologist case owners can request consultations from expert sub-specialists, who can be sent a link to a shared viewing session of a slide with the case owner [54]. The most obvious challenge faced in implementing deep learning in a clinical environment is the required technological infrastructure. The size of digitally scanned WSIs can be up to 3 gigabytes per slide, requiring an immense amount of storage space [55]. While the cost–benefit analysis has been shown to be worthwhile at large research centers, the high overhead costs may not be feasible for remote locations [56]. This can limit the amount of data available to be collected from under-represented regions, introducing further bias.

Additionally, the training of machine learning models requires graphics processing units (GPUs). While there is an increased availability of cloud-computing resources, their use introduces another bottleneck in the form of data transmission bandwidth [55]. Another factor to consider is that model training relies on the aggregation of large volumes of data that are typically only available through retrospective datasets; however, reliance on retrospective data with unknown inclusion/exclusion criteria can lead to unforeseen algorithmic biases [57]. These algorithmic biases can lead to automation bias, which is the inclination of humans to trust machine-generated information over their own intuition [58].

**Figure 2:**
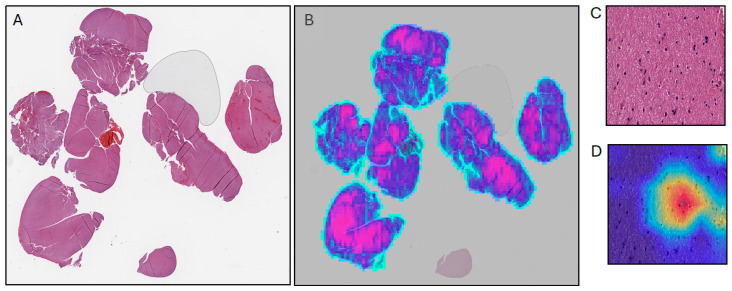
Examples of methods for deep learning explainability in digital pathology. (**A**) Thumbnail of FFPE WSI from TCGA-LGG dataset (**B**) Thumbnail from (**A**) with overlaid attention mapping from an attention-based MIL model. (**C**) Patch extracted at 40× magnification from (**A**,**D**) Grad-CAM activation mapping.

Model interpretability poses another meaningful barrier to implementation and efforts should be directed towards the development of explainable models for use by clinical practitioners [59]. Deep learning models can be difficult to interpret, given that feature selection is built into the training process. Two common methods used to try and decipher what parts of an image a model is using are attention [60] and activation mapping (e.g., Grad-CAM [61]). In digital pathology, attention is commonly used in a weakly supervised setting to select which patches are relevant for the end task. It is an integrated component of the model used during training. On the other hand, activation mapping is a post hoc technique used to examine the activations of different layers of a model. An example of attention weights and Grad-CAM activation maps can be seen in Figure 2.

Variation in the manual labeling of histopathologic images in the training of DL models presents a further challenge, due to interobserver variability in the evaluation of histopathologic images by pathologists [59]. For instance, there is a low concordance rate between pathologists in evaluating mitotic count, which remains a key determinant of histopathologic grade in various tumor entities, including, but not limited to, various gliomas and meningioma [62,63]. Finally, most DL models in neuro-oncology have been developed for use in glioma, given the limited availability of large, labeled datasets for rarer tumor types [46]. Synthetic pathology image generation, semi-supervised, weakly supervised, and self-supervised models hold promise to help overcome these challenges in the future.

Another important priority is the use of demographically inclusive datasets across key variables such as age, sex, gender, and ethnicity during model training, as imbalanced medical imaging training datasets have been shown to lead to models that perform worse in underrepresented groups [64,65]. For instance, a recent study using large public datasets from The Cancer Genome Atlas and the EBRAINS brain tumor atlas, comprised primarily of tumors from white patients, showed a 16.0% performance gap in the prediction of *IDH1* status in gliomas from black vs. white patients [65]. In addition to highlighting the need for bias mitigation strategies, including external validation with existing, prospective, and demographic-stratified datasets, the authors demonstrate the utility of novel strategies such as self-supervised vision foundation models to improve model generalizability.

## 8. Conclusions and Future Directions

Recent years have shown an explosive growth in AI development and deployment in a variety of settings, concurrent with rapid evolution in the field of diagnostic neuropathology. Brain cancer diagnoses have become increasingly complex and resource-intensive, as more molecular biomarkers are required to provide an integrative diagnosis to satisfy the current WHO classification scheme. This trend, while clinically justified, has created some degree of inequity between clinical laboratories in resource-rich and -poor countries. The use of DL in neuropathology, particularly the development of an image-based prediction of molecular driver workflow (e.g., *IDH1/2* mutation status or *CDKN2A/B* copy number status in low-grade gliomas), may provide accessible tools for pathology labs with limited access to molecular testing. This could be achieved, for instance, through the creation of a web-based server that can accept uploaded H&E images of low-grade infiltrating gliomas, taken with a dedicated microscope or even a mobile phone camera, and return a probability score of a genomic driver. Beyond direct applications to patient care, deep learning platforms may be used in the generation of synthetic images for education. While the deployment of AI tools in clinical, research, and educational contexts within neuropathology has been largely limited by the lack of training data to date, increasing global collaboration to assemble large, diverse, and publicly available datasets, coupled with the emergence of synthetic pathology, holds promise to help overcome these hurdles in the future.

## Figures and Tables

**Table 1:** Integrated classification of adult-type diffuse gliomas for diagnosis and grading.

		Adult-Type Diffusely Infiltrating Gliomas		
		Astrocytoma	Oligodendroglioma	Glioblastoma
Molecular classification for diagnosis	*IDH1/2*	Mutant	Mutant	Wildtype
	*1p19q*	Intact	Co-deleted	Intact
	*H3*	Wildtype	Wildtype	Wildtype
	Other	*ATRX, TP53* mutations	*TERTp, CIC, FUBP1, NOTCH1* mutations	*EGFR* amplification, *TERTp* mutation, +7/−10
Morphologic and molecular features for grading	WHO grade 2	Increased cellularity Nuclear atypia	Increased cellularity Nuclear atypia	N/A
	WHO grade 3	Elevated mitotic index	Elevated mitotic index MVP Necrosis	N/A
	WHO grade 4	MVP Necrosis *CDKN2A/B* HD ^a^	N/A	MVP ^b^ Necrosis ^b^

a—the presence of *CDKN2A/B* HD denotes a grade 4 astrocytoma, IDH-mutant, even in the absence of MVP and/or necrosis; b—if these high-grade histologic features are present in an IDH- and H3- wildtype adult-type diffuse glioma, this is sufficient for a diagnosis of glioblastoma, IDH-wildtype; however, these features are not required. If the tumor harbors *EGFR* amplification, *TERTp* mutation, and/or +7/−10, this is also indicative of glioblastoma, IDH-wildtype, even in a histologically low-grade tumor; HD—homozygous deletion; MVP—microvascular proliferation; N/A—not applicable; +7/−10—combined whole chromosome 7 gain and whole chromosome 10 loss.
